# Tunable Emission and Color Temperature of Yb^3+^/Er^3+^/Tm^3+^-Tridoped Y_2_O_3_-ZnO Ceramic Nano-Phosphors Using Er^3+^ Concentration and Excitation Pump Power

**DOI:** 10.3390/nano12122107

**Published:** 2022-06-19

**Authors:** Boxu Xu, Chao Song, Jie Song, Rui Huang, Zhenxu Lin, Yi Zhang, Shaomin Lin, Yanqing Guo, Guangxu Chen, Jun Song

**Affiliations:** 1 School of Materials Science and Engineering, Hanshan Normal University, Chaozhou 521041, China; 20190025@hstc.edu.cn (B.X.); songjie@hstc.edu.cn (J.S.); rhuang@hstc.edu.cn (R.H.); lzx2016@hstc.edu.cn (Z.L.); yee@hstc.edu.cn (Y.Z.); lsm678@hstc.edu.cn (S.L.); yqguo@hstc.edu.cn (Y.G.); cgx1312721921@sina.com (G.C.); 2 Key Laboratory of Optoelectronic Devices and Systems of Ministry of Education and Guangdong Province, College of Optoelectronic Engineering, Shenzhen University, Shenzhen 518060, China; songjun@szu.edu.cn; 3 State Key Laboratory of Advanced Technology for Materials Synthesis and Processing, Wuhan University of Technology, Wuhan 430070, China; 4 Chaozhou Branch of Chemistry and Chemical Engineering Guangdong Laboratory, Chaozhou 521041, China

**Keywords:** Y_2_O_3_, white light, adjustable color, sol–gel synthesis, nano-phosphor

## Abstract

In this study, a series of well-crystallized Yb^3+^/Er^3+^/Tm^3+^-tridoped Y_2_O_3_-ZnO ceramic nano-phosphors were prepared using sol–gel synthesis, and the phosphor structures were studied using X-ray diffraction, scanning electron microscopy, and thermogravimetric analysis. The phosphors were well crystallized and exhibited a sharp-edged angular crystal structure and mesoporous structure consisting of 270 nm nano-particles. All phosphors generated blue, green, and red emission bands attributed to Tm: ^1^G_4_→^3^H_6_, Er: ^2^H_11/2_ (^4^S_3/2_)→^4^I_15/2_, and Er: ^4^F_9/2_→^4^I_15/2_ radiative transitions, respectively. Increasing in luminescent centers, weakening of lattice symmetry, and releasing of dormant rare earth ions can enhance all emissions. Er^3+^ can obtain energy from Tm^3+^ to enhance green and red emission. These colors can be tuned by optimizing the doping concentrations of the Er^3+^ ion. The color coordinates were adjusted by tuning both the Er^3+^ concentration and excitation laser pump power to shift the color coordinates and correlated color temperature. The findings of this study will broaden the potential practical applications of phosphors.

## 1. Introduction

Due to 5*s*^2^5*p*^6^ shell shielding of the 4*f* electron layer, the trivalent lanthanide ion has abundant 4*f*^N^ energy levels which can realize radiative transitions of different wavelengths [1]. For example, Tb^3+^, Er^3+^, Ho^3+^, and Tm^3+^ are often used as activators to achieve upconversion (UC) luminescence [2]. The Er^3+^ ion has high luminescence efficiency and an emission peak located in the green and red light regions, which can be used as a source of green and red light [3]. As for the selection of sensitized ions, the Yb^3+^ ion is an efficient sensitizer for many rare earth (RE) elements because its absorption region is approximately 976–980 nm [4]. This ion has a large absorption cross section and can transfer absorbed infrared light to Ho^3+^, Tb^3+^, Pr^3+^, and Tm^3+^ through an energy transfer process. In addition, Er^3+^ plasma also achieves green and red emissions, which are conducive to wavelength regulation in different wavelength bands. Therefore, doped luminescence materials are widely made into phosphors [5], glass [6,7], ceramics [8], semiconductor crystal materials [9], and thin films [10], and these materials are used in many fields, such as conversion lasers [11], flat panel displays, biological probes [12], and solar cells [13].

Y_2_O_3_ has good performance as a matrix material, good chemical stability, high melting point, and desirable mechanical performance that allow this compound to be applied in challenging environments; in addition, the band gap width can accommodate most trivalent RE ion emission levels, and the radius of this compound and other RE ions are similar, leading to easy doping processes; finally, the low phonon energy of Y_2_O_3_ reduces the probability of no radiative transition and increases the probability of radiative transition [14]. These properties enable this compound to improve the luminescence efficiency of RE ions. Y_2_O_3_ is a type of RE oxide, and other RE elements that act as sensitizers and activators have the same valence state and similar oxide crystal structures that allows these other RE elements to mix easily into the lattice of Y_2_O_3_. These advantages make this compound a suitable substrate material. Similarly, ZnO has been widely used as an oxide matrix material in many fields. This compound is a multifunctional semiconductor material with a wide, direct band gap that is approximately 3.37 eV at room temperature. ZnO has three crystal structures, hexagonal wurtzite, sphalerite, and tetragonal halite, and the hexagonal wurtzite crystal structure is the most stable of these structures at room temperature. The density, surface work function, and relative molecular weight of hexagonal wurtzite ZnO are, respectively 5.606 g/cm^3^, 5.3 eV, and 81.39. The bonding state and geometric structure of ZnO crystals provide stable optical, chemical, and biological properties, as well as excellent thermal stability. ZnO materials have important applications as optical and infrared electric materials, indicative of its excellence as an oxide matrix [15]. Thus, many scholars have studied RE-doped Y_2_O_3_-ZnO composite matrix luminescent materials to enhance and adjust emissions. Mhlongo et al. synthesized Y_2_O_3_: Eu^3+^ and ZnO-Y_2_O_3_: Eu^3+^ nano-phosphors with different concentrations of Eu^3+^ using a sol–gel method. Their results show that the 612 nm red emission increases considerably, whereas green emission is suppressed when ZnO is added [16]. Danping Wang et al. synthesized Y_2_O_3_/ZnO UC films via a sol–gel method. The luminescence intensity of these films is enhanced significantly with a maximum value at an Er^3+^ doping concentration of 4 mol% [17]. Yuehui Tai et al. prepared Yb^3+^/Tm^3+^ co-doped Y_2_O_3_ UC materials and Y_2_O_3_:Yb^3+^, Tm^3+^/ZnO (Y/Z) composite photocatalysts for the photocatalytic degradation of dyes. The addition of Y_2_O_3_:Yb^3+^ and Tm^3+^ to ZnO substantially improves the absorption capacity for ultraviolet light, which enhances the photocatalytic activity [18]. However, the white luminescence process, optimum doping concentration of RE ions, and regulation of color temperature and coordinates still need further study.

Therefore, a series of Yb^3+^/Er^3+^/Tm^3+^-tridoped Y_2_O_3_-ZnO UC ceramic phosphors were prepared using sol–gel synthesis. Ceramic phosphor white light emission was mainly dominated by blue, green, and red emissions originating from Tm^3+^ and Er^3+^ transition mechanisms. Efficient color emission was attributed to Yb^3+^/Er^3+^/Tm^3+^ energy transfers. Additionally, the color emissions were tuned by changing the excitation laser pump power.

## 2. Materials and Methods

Yb_2_O_3_, Er_2_O_3_, Tm_2_O_3_, Y_2_O_3_, and ZnO were used as raw materials (Shanghai Aladdin Biochemical Technology Co., Ltd., Shanghai, China); these compounds were analytical grade (99.99% pure) and used without further purification.

A series of Yb^3+^/Er^3+^/Tm^3+^-tridoped Y_2_O_3_-ZnO UC ceramic nano-phosphors were prepared using typical sol–gel synthesis techniques. Initially, the RE oxides and Y_2_O_3_ and ZnO reagents were separately dissolved in specific volumes of nitric acid. The raw material proportions were as follows:

2.5 mol% Yb_2_O_3_ + *x*/2 mol% Er_2_O_3_ + 0.1 mol% Tm_2_O_3_ + (94.8 − *x*/2)/4 mol% Y_2_O_3_ + (94.8 − *x*/2)/2 mol% ZnO (where *x* = 0.2, 0.3, 0.4, 0.5, 0.6, 1.0, or 1.4 mol%).

Then, the Tm^3+^, Er^3+^, and Yb^3+^ nitric acid-based solutions were added to the Y^3+^- and Zn^2+^-containing solution, citric acid was added, and the mixed solution was then stirred and heated to obtain a precursor sol, which was aged at 24 °C for 24 h to form a gel. Afterward, the gel was annealed at 1200 °C for 2 h. The product was ground into a ceramic phosphor powder, which was subsequently characterized.

All analysis tests were carried out at room temperature. Ceramic phosphors’ Field-emission scanning electron microscopy (SEM) and morphology and energy dispersive spectroscopy (EDS) for the samples were carried out by a Hitachi S4800 FE-SEM (Hitachi Inc., Tokyo, Japan). The photos of high temperature microscope were taken by a HSML-FLEX-ODLT 1400 high temperature microscope (TA Instruments Inc., New Castle, DE, USA). The thermo-gravimetric analysis (TG) analysis of the samples was performed from 25 to 700 °C using a TA STA 409PC thermal analyzer (TA Instruments Inc., New Castle, DE, USA). The crystal structure and phase purity were analyzed from 5 to 90° by a Bruker D8 (Bruker Inc., Karlsruhe, Germany) Discover X-ray powder diffractometer (XRD) with a nickel-filtered Cu-Kα radiation (λ = 1.5406 Å). The grain size was measured by a dynamic laser scattering (DLS) test with a BT-9300Z laser particle size distributor (Bettersize Inc., Dandong, China). The photoluminescence (PL) spectrum was recorded by using a FLS 1000, Edinburgh instruments fluorescence spectrometer (Edinburgh instruments Inc., Livingston, UK) under a MSI 980 nm laser diode (MSI Inc., Taipei, China).

## 3. Results

### 3.1. SEM Morphology and EDS Mapping

**Figure 1:**
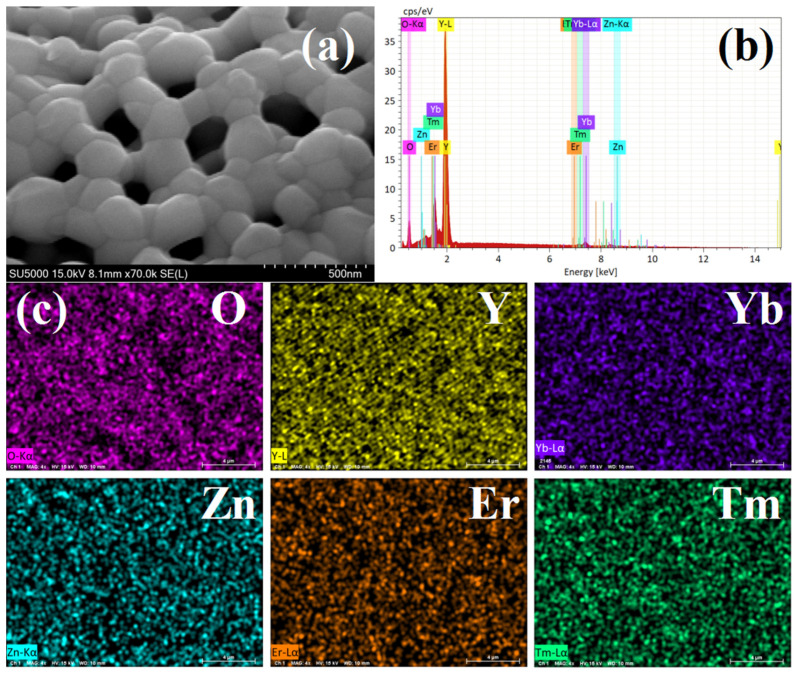
(**a**) SEM morphology (Er^3+^: 1 mol%) (**b**) EDS spectrogram of representative Y_2_O_3_-ZnO:Yb^3+^/Er^3+^/Tm^3+^ nano-phosphor (Er^3+^: 1 mol%) (**c**) EDS mapping for each element.

The nano-phosphor material surface morphology was characterized using SEM. Figure 1a shows a representative SEM image of the Yb^3+^/Er^3+^/Tm^3+^-tridoped Y_2_O_3_-ZnO ceramic nano-phosphor (Er^3+^: 1 mol%), clearly indicating the crystal size variation. As shown in Figure 1a, the samples were well crystallized and exhibited a sharp-edged angular crystal structure and mesoporous structure consisting of smaller nano-particles. Furthermore, the nano-phosphor chemical composition was analyzed using EDS maps, as shown in Figure 1b,c. Clearly, the nano-phosphor contained O, Y, Tm, Er, Yb, and Zn. No impurities were detected.

Moreover, the semi-quantitative proportional variation in elements is also obtained by EDS and is shown in Table 1. It can be seen that the proportion of each element is basically consistent with the experimental design.

### 3.2. XRD Results

**Figure 2:**
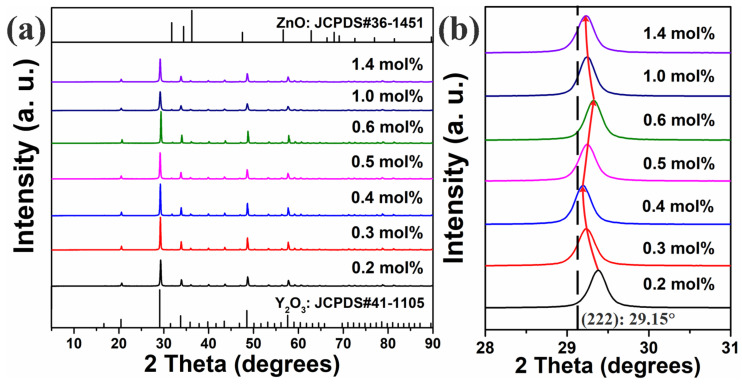
(**a**) Sample XRD patterns and (**b**) corresponding (222)-plane peak shifts.

Figure 2a shows the XRD patterns of all the samples. The diffraction peaks were sharp, indicating that all the samples exhibited good crystallinity. With increasing Er^3+^ concentration, no additional peaks appeared in any of the spectra. According to the Joint Committee on Powder Diffraction Standards (JCPDS), the main diffraction peaks were indexed to the characteristic peaks of a Y_2_O_3_ body centered cubic structure (JCPDS#41-1105). In addition, weak ZnO characteristic peaks (JCPDS#36-1451) also appeared in each pattern. The three principal diffraction peaks of ZnO overlapped with a diffraction peak of Y_2_O_3_, thus the diffraction peaks of ZnO were unclear in the spectrograms. Additionally, none of the patterns exhibited any peaks attributed to other phases, indicating that both Y_2_O_3_ and ZnO were independent.

To further elucidate how the Er^3+^ concentration affected the matrix lattice, the main Y_2_O_3_ (222) crystal plane diffraction peaks were amplified, as shown in Figure 2b. With increasing Er^3+^ concentration, the (222) peak first shifted to a lower angle. Then, as the Er^3+^ concentration increased to 0.4 mol%, the (222) peak shifted to a higher angle. Above 0.6 mol%, further increasing the Er^3+^ concentration shifted the (222) peak to a lower angle again. According to Bragg’s law, 2*d*sin*θ* = *nλ* (where *d* is the interplanar crystal spacing, *θ* is the angle between the incident X-ray and crystal face, *n* is the diffraction order, and *λ* is the X-ray wavelength), and lattices expand when the diffraction peak shifts to a lower angle, and vice versa. Moreover, Er_2_O_3_ and Y_2_O_3_ have almost identical lattice structures and the Y_2_O_3_ lattice gap lacks the space to accommodate Er^3+^ ions, thus Y^3+^ ions can only be substituted by Tm^3+^ ones. Er^3+^ (0.89 Å) has a smaller ionic radius than Y^3+^ (0.90 Å), thus the host lattice Y_2_O_3_ part shrank and the (222) peak shifted to a higher angle when Y_2_O_3_ was doped with Er^3+^ ions. As shown in Figure 2b, with increasing Er^3+^ concentration, the local lattice Y_2_O_3_ Er^3+^ concentration initially gradually decreased. Then, as the Er^3+^ concentration increased from 0.4 to 0.6 mol%, the local lattice Y_2_O_3_ Er^3+^ concentration considerably increased. Above 0.6 mol%, further increasing the Er^3+^ concentration decreased the local lattice Y_2_O_3_ Er^3+^ concentration again. Because the host lattice consisted of both Y_2_O_3_ and ZnO parts, increasing the Er^3+^ concentration from 0.2 to 0.4 mol% initially decreased the Er^3+^ local concentration in the ZnO lattice, but this local concentration increased when the Er^3+^ concentration was in the range from 0.4 to 0.6 mol%; subsequently, this local concentration increased again when the Er^3+^ concentration was above 0.6 mol%.

The average crystallite size could be calculated with the Scherrer formula:D = kλ/(βcosθ), where D is the crystallite grain size of the nano-crystals, λ is the X-ray wavelength (0.154056 nm), θ is the Bragg angle of the diffraction peak, k is the Scherrer constant that is conventionally set to be 0.89, and β is the corrected full width at half maximum (FWHM) of the main diffraction characteristic peak of the XRD pattern. Table 2 lists the average crystallite sizes of the samples. The results show that the average crystallite sizes of samples vary slightly with Er^3+^ concentration increase. The average crystallite sizes of nano-phosphors are about 270 nm

**Figure 3:**
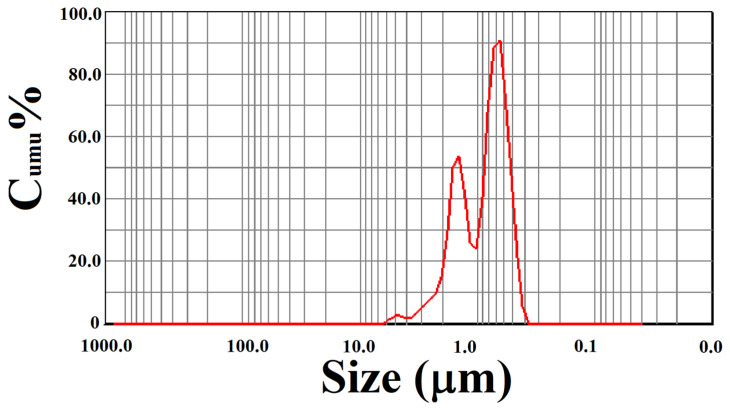
DLS patterns of sample (Er^3+^: 0.6 mol%).

In order to investigate grain size and agglomeration, a DLS measurement is done for the sample (Er^3+^: 0.6 mol%) which is finely ball milled for 3 h, and the spectra is shown in Figure 3. The results show that grain sizes range from 300 nm to 6000 nm.

The information of DLS test for the sample (Er^3+^: 0.6 mol%) are list in Table 3. It can be seen that the median size of grain is 872 nm, and the average grain size is 1185 nm. Conspicuously, the particles aggregate into large porous grains, which is consistent with the observation of SEM imagine. This indicates that most phosphor particles are maintained a stable porous structure in nanoscale, only a few particles remain independent.

### 3.3. Photoluminescence (PL) Properties

**Figure 4:**
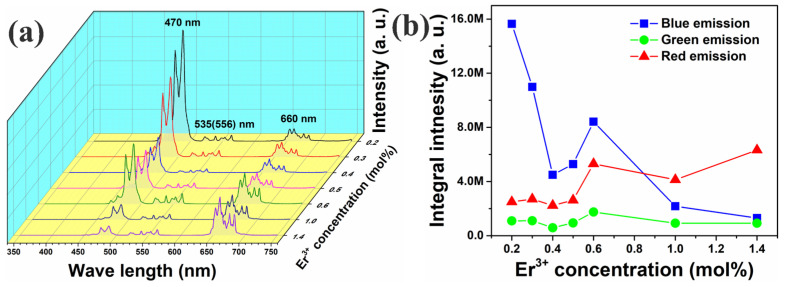
(**a**) UC emission spectra generated for Y_2_O_3_-ZnO:Yb^3+^/Er^3+^/Tm^3+^-tridoped nano-phosphors excited using 980 nm laser diode operated at 1.0-W pump power. (**b**) Blue, green, and red peak intensities of UC spectra plotted as functions of Er^3+^ concentration.

Figure 4a shows the sample PL spectra. Each PL spectrum exhibited blue, green, and red emission bands in ranges of 460–490, 510–570, and 630–680 nm, respectively. The emission peaks centered at approximately 470, 535(556), and 660 nm were attributed to the Tm^3+^ ion ^1^G_4_→^3^H_6_, Er^3+^ ion ^2^H_11/2_ (^4^S_3/2_)→^4^I_15/2_, and Er^3+^ ion ^4^F_9/2_→^4^I_15/2_ energy-level transitions, respectively. Figure 4b shows blue (460–490 nm), green (510–570 nm), and red emission (630–680 nm) integral intensities plotted as functions of Er^3+^ concentration. As the Er^3+^ concentration increased, the blue emission initially intensified dramatically. As the Er^3+^ concentration increased to 0.4 mol%, the emissions increased to a peak at an Er^3+^ concentration of 0.6 mol%, then decreased. In contrast, both green and red emissions initially intensified, but subsequently weakened in a small range, with the peak appearing at 0.3 mol%. Increasing the Er^3+^ concentration to 0.4 mol% resulted in both emissions increasing again to another peak at an Er^3+^ concentration of 0.6 mol%. After another decline in emission from 0.6 mol% to 1.0 mol%, emissions increased again. Additionally, the blue emission was stronger than the red one at low Er^3+^ concentrations. However, at Er^3+^ concentrations above 1.0 mol%, the red emission was stronger than the blue one.

**Figure 5:**
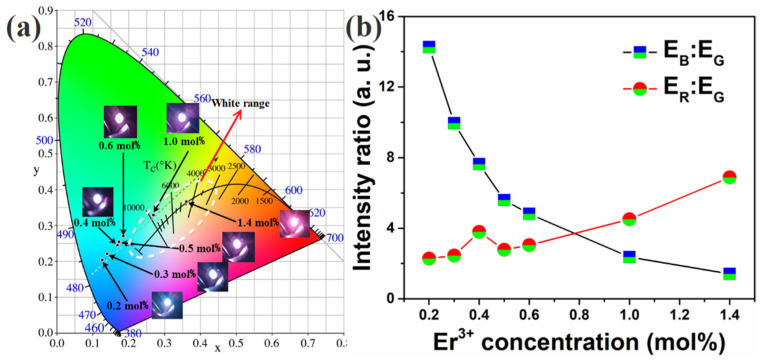
(**a**) CIE chromaticity coordinates for Y_2_O_3_-ZnO: Yb^3+^/Er^3+^/Tm^3+^ nano-phosphors under 1.0 W-980 nm diode laser excitation with different Er^3+^ doping concentrations. (**b**) The intensity ratios of blue emission to green emission and red emission to green emission.

The International Commission on Illumination (internationale de I’èclairage, CIE) chromaticity test was performed, and the luminescence photos and corresponding results are shown in Figure 5a. The coordinates of samples were approximately linear with wide dispersion. As the Er^3+^ concentration increased, the color of fluorescence changed from white to blue. Figure 5b shows the ratios of blue emission to green emission (E_B_/E_G_) and red emission to green emission (E_R_/E_G_). As the Er^3+^ concentration increased, the E_B_/E_G_ ratio decreased gradually, whereas the E_R_/E_G_ ratio increased, resulting in color-tunable emission by adjusting the Er^3+^ concentrations. The reduced blue emission and increased green emission jointly determined how the color coordinates changed to the white region with the increase in Er^3+^ doping concentration. Green emission had a weak effect on the movement of color coordinates because of its weak relative intensity.

**Figure 6:**
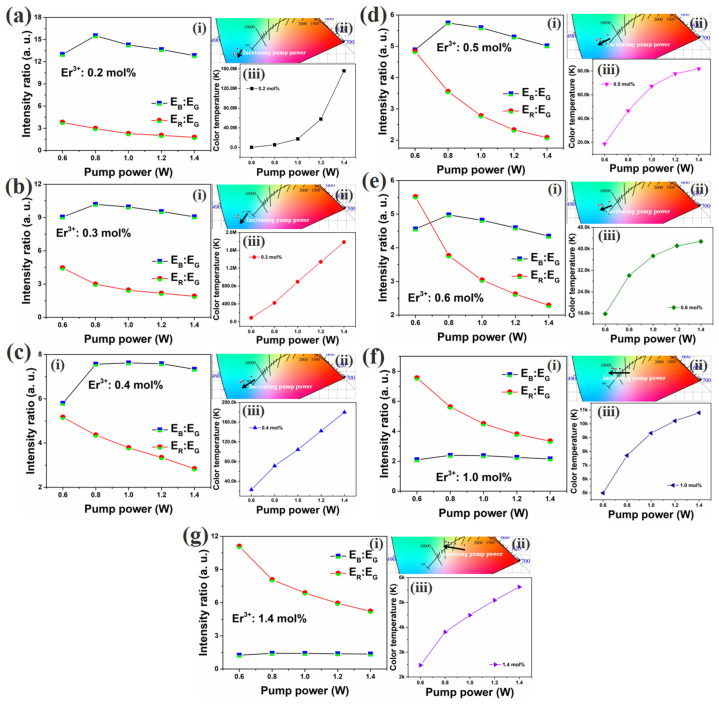
The intensity ratios of blue emission to green emission and red emission to green emission (i), CIE chromaticity coordinates (ii) and CCT for Y_2_O_3_-ZnO:Yb^3+^/Er^3+^/Tm^3+^-tridoped nano-phosphors (iii) for Y_2_O_3_-ZnO: Yb^3+^/Er^3+^/Tm^3+^ nano-phosphors under 980 nm diode laser excitation with different pump powers (0.6, 0.8, 1.0, 1.2, and 1.4 W); (**a**) Er^3+^: 0.2 mol%, (**b**) Er^3+^: 0.3 mol%, (**c**) Er^3+^: 0.4 mol%, (**d**) Er^3+^: 0.5 mol%, (**e**) Er^3+^: 0.6 mol%, (**f**) Er^3+^: 1.0 mol%, and (**g**) Er^3+^: 1.4 mol%.

Nano-phosphors cannot always be replaced in practice. Therefore, changing the color of luminescence must be accomplished through other ways. Changing the power of laser excitation is a more convenient method to adjust the color coordinates in practical operation. Therefore, CIE chromaticity coordinates for Y_2_O_3_-ZnO: Yb^3+^/Er^3+^/Tm^3+^ nano-phosphors under 980 nm diode laser excitation with different pump powers which were 0.6, 0.8, 1.0, 1.2, and 1.4 W were measured, as shown in (ii) of Figure 6. The color coordinates shifted to the blue direction as the laser power increased when the Er^3+^ doping concentration ranged from 0.2 to 0.6 mol%, as shown in (ii) of Figure 6a–e. When the Er^3+^ doping concentration was greater than 0.6 mol%, an increase in laser power resulted in color coordinates shifting to green as shown in (ii) of Figure 6f,g. As shown in Figure 5, it is known that the position of color coordinates is related to the emission intensity ratio of blue to green and red to green, and the intensity ratios of blue emission to green emission and red emission to green emission are shown in (i) of Figure 6. it can be seen that the Er^3+^ doping concentration range had a larger ratio of E_B_/E_G_ than that of E_R_/E_G_. Increasing the laser power widened the difference between the E_B_/E_G_ and E_R_/E_G_ ratios.

The correlated color temperature (CCT) of each sample were calculated according to the color coordinates, and the results are shown in (iii) of Figure 6. As the Er^3+^ doping concentration increased, the CCT decreased. As shown in (iii) of Figure 6a, due to the intense blue emission, the CCT increased almost exponentially as the laser power increased when the Er^3+^ doping concentration was low (0.2 mol%). Then, in the range of Er^3+^ doping concentrations from 0.3 to 0.4 mol%, the CCT increased linearly with increasing laser power, as shown in (iii) of Figure 6b,c. When the Er^3+^ doping concentration was above 0.4 mol%, the CCT still increased with increasing laser power, but the rate of increase was slower, as shown in (iii) of Figure 6d–g.

**Figure 7:**
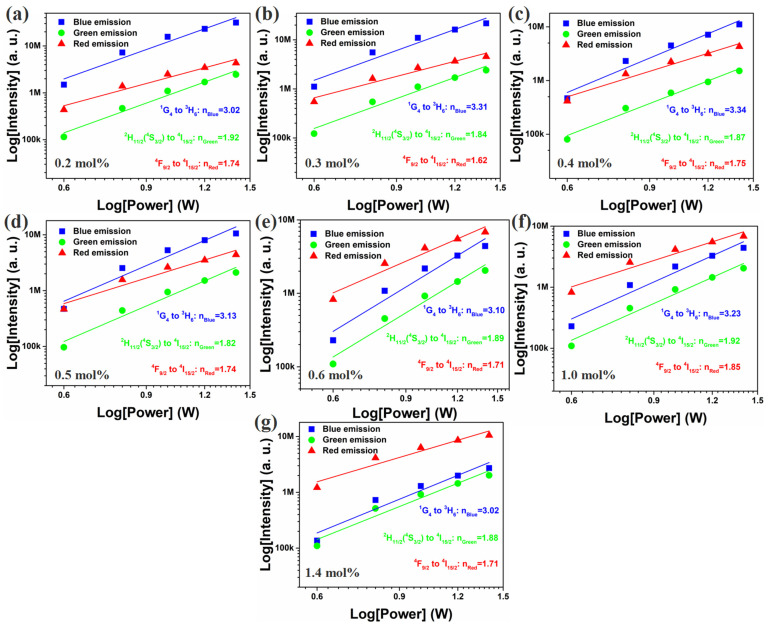
Log(emission intensity) plotted as functions of Log(laser diode pump power) for Y_2_O_3_-ZnO:Yb^3+^/Er^3+^/Tm^3+^-tridoped nano-phosphors (Er^3+^: 0.6 mol%); (**a**) Er^3+^: 0.2 mol%, (**b**) Er^3+^: 0.3 mol%, (**c**) Er^3+^: 0.4 mol%, (**d**) Er^3+^: 0.5 mol%, (**e**) Er^3+^: 0.6 mol%, (**f**) Er^3+^: 1.0 mol%, and (**g**) Er^3+^: 1.4 mol%.

Luminescence intensity, I*_UC_*, follows the relation I*_UC_*∝P*^n^_pump_*, where *n* is the number of photons required to populate the emitting state [19]. The plot of I*_UC_* versus P*_pump_* with a double logarithmic scale for Y_2_O_3_-ZnO: Yb^3+^/Er^3+^/Tm^3+^ nano-phosphors are shown in Figure 7. The values of *n* for blue emission are 3.02, 3.31, 3.34, 3.13, 3.10, 3.23 and 3.02, respectively. The values of green emission are 1.92, 1.84, 1.87, 1.82, 1.89, 1.92 and 1.88, respectively. The values of red emission are 1.74, 1.62, 1.75, 1.74,1.71, 1.85 and 1.71, respectively. The results indicate that blue emission involves a three-photon process, and green and red emission involve a two-photon process, and the change of Er^3+^ doping concentration has no obvious effect on emission processes.

## 4. Discussion

**Figure 8:**
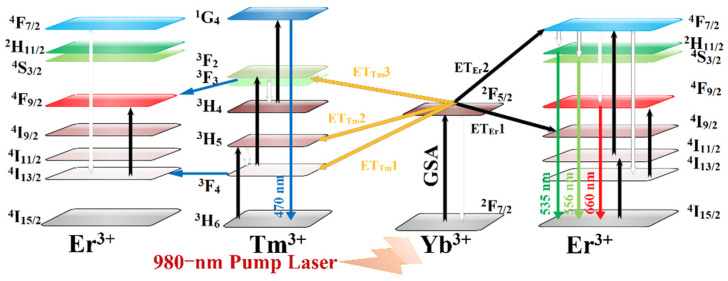
Y_2_O_3_-ZnO:Yb^3+^/Er^3+^/Tm^3+^-tridoped nano-phosphor UC energy transfer mechanisms.

For a better understanding of the energy transfer among the Yb^3+^ Er^3+^ and Tm^3+^, the energy-level diagrams of Yb^3+^, Er^3+^, and Tm^3+^ ions are shown in Figure 8. First, Yb^3+^ absorbs energy from the 980 nm pump laser [20,21], Yb: ^2^F_7/2_ + 980 nm laser→ Yb: ^2^F_5/2_; then, energy is transferred from Yb^3+^ to Tm^3+^, and the process is described by the following equations [22,23]:

Yb: ^2^F_5/2_ + Tm: ^3^H_6_→Tm: ^3^H_5_ (ET_Tm_1),

Tm: ^3^H_5_ (nonradiative)→ Tm: ^3^F_4_,

Yb: ^2^F_5/2_ + Tm: ^3^F_4_→Tm: ^3^F_2_(^3^F_3_) (ET_Tm_2),

Tm: ^3^F_2_(^3^F_3_) (nonradiative)→Tm: ^3^H_4_,

Yb: ^2^F_5/2_ + Tm: ^3^H_4_→Tm: ^1^G_4_ (ET_Tm_3),

Tm: ^1^G_4_→Tm: ^3^H_6_ + blue emission.

Another energy transfer path is from Yb^3+^ to Er^3+^, which is described as follows [24,25]:

Yb: ^2^F_5/2_ + Er^3+^: ^4^I_15/2_→Er^3+^: ^4^I_11/2_ (ET_Er_1),

Yb: ^2^F_5/2_ + Er: ^4^I_11/2_→Yb: ^2^F_7/2_ + Er: ^4^F_7/2_ (ET_Er_2),

Er: ^4^F_7/2_ (nonradiative)→Er: ^4^H_11/2_(^4^S_3/2_),

Er: ^4^H_11/2_(^4^S_3/2_)→^4^I_15/2_ + green emission.

In addition,

Er: ^4^F_7/2_ (nonradiative)→Er: ^4^F_9/2_;

And

Er: ^4^I_11/2_ (nonradiative)→ Er: ^4^I_13/2_:

Er: ^4^I_13/2_ + Yb: ^2^F_5/2_→Er: ^4^F_9/2_ (ET_Er_3),

Er: ^4^F_9/2_→^4^I_15/2_ + red emission.

Therefore, blue emission can be attributed to the transition of Tm: ^1^G_4_→^3^H_6_; green emission can be attributed to the transition of ^4^H_11/2_(^4^S_3/2_)→^4^I_15/2_; and red emission can be attributed to the transition of Er: ^4^F_9/2_→^4^I_15/2_. As shown in Figure 4b, the spectra of green and red emissions had two peaks. For the first peak, blue emission was sharply reduced in this range. It is obvious that the energy is also transferred between Tm^3+^ and Er^3+^. according to the research of D. Yan et al., the transition process between Tm^3+^ and Er^3+^ can be described by the following equations [26,27,28,29]:

Er: ^4^I_11/2_ + Tm: ^3^H_5_ → Er: ^4^S_3/2_ + Tm: ^3^H_6_,

Er: ^4^I_9/2_ + Tm: ^3^F_4_ → Er: ^4^S_3/2_ + Tm: ^3^H_6_,

Er: ^4^I_13/2_ + Tm: ^3^H_4_ → Er: ^4^H_11/2_ + Tm: ^3^H_6_,

Er: ^4^I_13/2_ + Tm: ^3^H_5_ → Er: ^4^F_9/2_ + Tm: ^3^H_6_.

Contrary to Adnan Khan’s research, Er^3+^ not only cause the quenching of Tm^3+^ [30], but also receives energy from Tm^3+^ (Er: ^4^I_13/2_ + Tm: ^3^H_5_ → Er: ^4^F_9/2_ + Tm: ^3^H_6_), explaining the small increase in green and red emissions. When the Er^3+^ doping concentration was 0.6 mol%, three emissions exhibited a strong peak, possibly because of the increase in luminescent centers, weakening of lattice symmetry, and release of dormant RE ions located in the symmetric positions of the Y_2_O_3_ lattice [31,32]. Increasing the Er^3+^ concentration up to 1.4 mol% resulted in emission enhancement, and the enhancement of the green and red emissions should be attributed to the energy from Tm^3+^. Meanwhile, it also can be seen Figure 4b that compared with the green and red emission intensities of sample of which Er^3+^ doping concentration is 0.6 mol%, those of which Er^3+^ doping concentration is 1.0 mol% begin to decrease, which can be attributed to the concentration quenching of Er^3+^. When Er^3+^ doping concentration is large and the distance between the centers is less than the critical distance, they produce cascade energy transfer, i.e., from one center to the next, and then to the next until it finally enters a quenching center, resulting in the quenching of luminescence.

## 5. Conclusions

A series of Yb^3+^/Er^3+^/Tm^3+^ tri-doped Y_2_O_3_-ZnO ceramic nano-phosphors were prepared via a sol–gel method. The luminescence and structure of the obtained phosphors were investigated. Ceramic nano-phosphors were well crystallized and exhibited a sharp-edged angular crystal structure and mesoporous structure consisting of smaller particles which size were about 270 nm. As described in the results, the blue emission band at 470 nm, green emission band at 535 nm, and red emission band at 660 nm are attributed to the ^1^G_4_ to ^3^H_6_ energy level transitions of Tm^3+^, ^2^H_11/2_ (^4^S_3/2_) to ^4^I_15/2_ radiative transitions of Er^3+^_,_ and ^4^F_9/2_ to ^4^I_15/2_ radiative transitions of Er^3+^, respectively. Er^3+^ can get energy from Tm^3+^ to enhance green and red emission. Yb^3+^, Er^3+^, and Tm^3+^ did not mediate any obvious change in the crystal structure of either Y_2_O_3_ or ZnO matrix. The color coordinates were adjusted by changing the Er^3+^ doping concentration and laser power, and the emission color was tuned to white light indicating the practical applications of the prepared phosphor in display devices and lasers. Under different doping concentrations, the CCT was adjusted in different ranges by changing the power of the excited laser. The energy transfer of Tm^3+^ to Er^3+^, increase in luminescent centers, and release of Y_2_O_3_ symmetrically dormant RE ions are the fundamental reasons for the emissions change.

## Figures and Tables

**Table 1:** Chemical element composition ratio obtained by EDS spectrum.

Er^3+^ Concentration (mol%)		0.2	0.3	0.4	0.5	0.6	1.0	1.4
**Atom (%)**	**Y**	24.28	23.19	24.89	22.01	24.22	23.81	24.25
	**Zn**	20.35	19.86	20.31	20.89	19.28	20.22	19.77
	**O**	51.79	53.26	51.14	53.13	52.44	51.41	51.12
	**Yb**	3.21	3.19	3.02	3.32	3.22	3.18	3.33
	**Tm**	0.18	0.18	0.22	0.16	0.21	0.17	0.20
	**Er**	0.19	0.32	0.42	0.49	0.63	1.21	1.33

**Table 2:** The average crystallite sizes of nano-phosphors.

Er^3+^ Concentration (mol%)	0.2	0.3	0.4	0.5	0.6	1.0	1.4
Crystallite size (nm)	261	278	257	275	277	280	256

**Table 3:** Information of DLS test for the sample (Er^3+^: 0.6 mol%).

Er^3+^ Concentration (mol%)	Median Size (nm)	Average Grain Size (nm)	Fitting Residual Error
0.6	872	1185	0.095%

## Data Availability

The data presented in this study are available on request from the corresponding author.
